# Diagnostic and prognostic significance of mast cell markers in HIV/AIDS: Current insights and future directions

**DOI:** 10.1097/MD.0000000000038117

**Published:** 2024-05-17

**Authors:** Emmanuel Ifeanyi Obeagu

**Keywords:** AIDS, diagnosis, HIV, mast cell markers, prognostic significance

## Abstract

Human immunodeficiency virus (HIV) infection continues to pose significant global health challenges, necessitating advancements in diagnostic and prognostic approaches to optimize disease management. While primarily recognized for their roles in allergic responses, mast cells have emerged as potential markers with diagnostic and prognostic significance in the context of HIV/AIDS. This paper aims to synthesize current insights and delineate future directions regarding the utility of mast cell markers in diagnosing HIV infection, predicting disease progression, and guiding therapeutic strategies. Mast cells, equipped with distinct markers such as tryptase, chymase, carboxypeptidase A3, and c-kit/CD117 receptors, exhibit tissue-specific expression patterns that offer potential as diagnostic indicators for HIV infection. Understanding the dynamics of these markers in different tissues and body fluids holds promise for accurate HIV diagnosis, disease staging, and monitoring treatment responses. Moreover, the prognostic significance of mast cell markers in HIV/AIDS lies in their potential to predict disease progression, immune dysregulation, and clinical outcomes. The integration of mast cell markers into clinical applications offers promising avenues for refining diagnostic assays, patient monitoring protocols, and therapeutic strategies in HIV/AIDS. Future research directions involve the development of novel diagnostic tools and targeted therapies based on mast cell-specific markers, potentially revolutionizing clinical practice and enhancing patient care in the management of HIV/AIDS. Continued investigations into mast cell markers’ diagnostic and prognostic implications hold immense potential to advance our understanding and improve outcomes in HIV/AIDS management.

## 1. Introduction

Human immunodeficiency virus (HIV) infection remains a profound global health concern, necessitating precise diagnostic tools and prognostic indicators to optimize disease management and therapeutic interventions.^[1]^ Amidst the intricate landscape of immune responses, mast cells have garnered attention for their potential diagnostic and prognostic significance in HIV/AIDS.^[2]^ This paper aims to provide a comprehensive overview of the current understanding, challenges, and future prospects surrounding the utility of mast cell markers in diagnosing HIV infection, predicting disease progression, and guiding therapeutic approaches. Mast cells, traditionally acknowledged for their involvement in allergic reactions and host defense against parasites, have exhibited multifaceted roles in immune responses beyond their conventional functions.^[3]^ Recent research has unveiled their capacity to act as sentinels in various tissues, expressing unique markers such as tryptase, chymase, carboxypeptidase A3, and c-kit/CD117 receptors.^[4,5]^ These markers, identified in different tissue microenvironments and body fluids, present intriguing prospects as potential diagnostic tools for HIV infection.^[6]^ Moreover, beyond their diagnostic implications, the prognostic value of mast cell markers in HIV/AIDS represents a burgeoning area of investigation. The intricate interplay between mast cell activation, tissue distribution, and their association with disease severity offers potential insights into predicting disease progression, immune dysregulation, and clinical outcomes in individuals living with HIV/AIDS.^[7]^

Understanding mast cell-mediated immune responses in the context of HIV infection holds promise for elucidating their role as both diagnostic and prognostic indicators. The exploration of mast cell activation patterns, their interactions within the immune milieu, and their potential influence on viral replication and immune activation forms a critical aspect of their significance in HIV/AIDS pathogenesis.^[8]^ As the field progresses, integrating mast cell markers into clinical applications may offer avenues for refining diagnostic assays, designing personalized treatment strategies, and predicting therapeutic responses in individuals affected by HIV/AIDS. However, challenges persist in comprehensively understanding the functional nuances and standardizing the use of mast cell markers in clinical practice. This paper aims to synthesize the current state of knowledge regarding mast cell markers in HIV/AIDS, addressing their diagnostic potential, prognostic implications, and their role in immune responses. Moreover, it delineates future research directions and challenges in harnessing the full potential of mast cell markers as indispensable tools for diagnosing, prognosticating, and managing HIV/AIDS. Continued research endeavors hold the promise of advancing our understanding and clinical utilization of mast cell markers, potentially revolutionizing the approach to HIV/AIDS diagnosis, prognosis, and therapeutic interventions.

## 2. Mast cells

Mast cells are a type of immune cell primarily recognized for their involvement in allergic responses and immune defense against parasites. These cells are found in various tissues throughout the body, particularly near blood vessels and nerves, where they play pivotal roles in the immune system’s immediate response to pathogens and tissue injury.^[7]^ Key features of mast cells include their distinctive granules containing bioactive molecules like histamine, proteases (such as tryptase and chymase), cytokines, chemokines, and growth factors.^[8,9]^ Upon activation through various stimuli, mast cells rapidly release these granule-stored compounds into the surrounding tissue, triggering inflammatory and immune responses.^[10]^ One of the most well-known functions of mast cells is their contribution to allergic reactions. When an individual with allergies encounters an allergen, such as pollen or certain foods, mast cells recognize the allergen through specific receptors (e.g., immunoglobulin E [IgE] receptors) on their surface. This recognition prompts mast cell activation, leading to the release of histamine and other inflammatory mediators, causing symptoms like itching, swelling, and respiratory distress.^[11]^ Beyond allergies, mast cells actively participate in immune surveillance and defense. They interact with other immune cells, such as T cells, B cells, and dendritic cells, influencing the immune response. Mast cells also contribute to wound healing, tissue repair, and protection against certain infections, particularly parasitic infections.^[12]^ In addition to their classical roles in allergy and host defense, recent research has unveiled the involvement of mast cells in various diseases and conditions, including autoimmune disorders, cardiovascular diseases, cancer, and neuroinflammatory conditions. Their ability to modulate the immune response, release a diverse array of mediators, and influence surrounding cells positions mast cells as intriguing targets for therapeutic interventions across multiple medical fields.^[13]^ The intricate functions of mast cells and their contributions to both health and disease continue to be the subject of extensive research. Understanding the complexities of mast cell biology offers potential avenues for developing novel therapeutic strategies and advancing treatments for various immune-mediated and inflammatory conditions.

## 3. Roles of mast cells in immunity

Mast cells play diverse and pivotal roles in the immune system, contributing significantly to both innate and adaptive immunity.^[14]^ Mast cells are well-known for their involvement in allergic reactions. Upon exposure to allergens, such as pollen, food, or insect venom, mast cells release a cascade of mediators, notably histamine, leukotrienes, prostaglandins, and cytokines. These mediators cause the typical allergic symptoms, including itching, sneezing, swelling, and in severe cases, anaphylaxis.^[15,16]^ Mast cells are strategically located near blood vessels and mucosal surfaces, acting as sentinels against invading pathogens. They contribute to the first line of defense by recognizing and responding to pathogens through pattern recognition receptors. Activation triggers the release of antimicrobial substances, cytokines, and chemokines, recruiting other immune cells to the site of infection.^[14]^ Mast cells are critical in initiating and regulating inflammatory responses. They release various mediators that promote inflammation, leading to increased blood flow, vascular permeability, and recruitment of immune cells to the site of injury or infection. Additionally, mast cells play a role in tissue repair and wound healing by releasing factors that promote tissue growth and remodeling.^[16]^ Mast cells interact with other immune cells, including T cells, B cells, and dendritic cells, influencing adaptive immune responses. They can regulate T cell activation and differentiation, modulate antibody production by B cells, and participate in the regulation of immune tolerance and memory responses.^[15]^ Mast cells release factors that contribute to angiogenesis (formation of new blood vessels) and increased vascular permeability, which are crucial processes during tissue repair, wound healing, and inflammatory responses.^[14]^ Mast cells interact with nerve fibers and participate in neuro-immune crosstalk. They release substances that can activate nerve endings, contributing to sensory processes, neurogenic inflammation, and certain neurological disorders.^[15]^ In addition to their pro-inflammatory roles, mast cells also exhibit regulatory functions. They contribute to immune homeostasis by secreting anti-inflammatory mediators and cytokines that can downregulate immune responses and maintain tissue equilibrium.

## 4. Mast cell markers and diagnostic utility

Mast cells possess specific markers that have potential diagnostic utility in various medical conditions, including allergic diseases, autoimmune disorders, and certain malignancies. These markers aid in identifying and characterizing mast cells in different tissues and body fluids, contributing to diagnostic assessments and disease monitoring.^[17,18]^ Tryptase, a protease stored in mast cell granules, is 1 of the most widely used markers for mast cell identification. Elevated levels of tryptase in serum or tissues are indicative of mast cell activation and are often measured to diagnose systemic mastocytosis, a rare disorder characterized by abnormal mast cell proliferation.^[19,20]^ Another protease stored in mast cell granules, chymase, is often measured alongside tryptase in diagnosing mastocytosis.^[21]^ The ratio of tryptase to chymase levels can help differentiate between different forms of mastocytosis and assess disease severity.^[22]^ Mast cells express the c-kit/CD117 receptor on their surface, which plays a crucial role in their development, survival, and activation. Immunohistochemical staining for c-kit/CD117 is used to identify mast cells in tissue samples, aiding in the diagnosis of mastocytosis and other mast cell-related disorders.^[23,24]^ Carboxypeptidase A3 is a mast cell-specific enzyme that can be used as an additional marker for mast cell identification in tissues. Elevated levels of carboxypeptidase A3 are associated with mast cell activation and might serve as a diagnostic indicator in certain mast cell-related conditions.^[25,26]^ Besides the aforementioned markers, mast cells express various surface receptors, including FcεRI (the high-affinity IgE receptor) and CD203c. These receptors are often utilized in flow cytometry-based assays to identify and quantify mast cells in blood or other body fluids, aiding in diagnosing allergic conditions and monitoring treatment responses.^[27,28]^ Utilizing mast cell markers in diagnostic assessments allows for the identification, localization, and quantification of mast cells in different tissues or body fluids, contributing to the diagnosis and management of various mast cell-related disorders. The measurement of these markers provides valuable insights into mast cell activation, differentiation, and involvement in pathological conditions, facilitating tailored treatment strategies and disease monitoring.^[29]^

## 5. Prognostic significance of mast cell markers

Mast cell markers have shown promise as potential prognostic indicators in several medical conditions, reflecting disease severity, progression, and treatment responses. The prognostic significance of mast cell markers is particularly evident in various diseases and disorders.^[30]^ In certain cancers, such as gastrointestinal stromal tumors and melanoma, the density of mast cells within the tumor microenvironment has been linked to prognosis. High mast cell infiltration in tumors has shown associations with improved outcomes, including better survival rates and reduced risk of metastasis in some cases.^[31]^ Mast cell activation in cardiovascular diseases, including atherosclerosis and heart failure, has been associated with adverse outcomes.^[32]^ Elevated levels of mast cell-derived mediators, such as tryptase and chymase, have shown correlations with increased inflammation, plaque instability, and adverse cardiac remodeling, potentially serving as prognostic markers for cardiovascular events.^[33]^ Mast cell activation and mediator release are central to allergic reactions. In allergic diseases like asthma and allergic rhinitis, increased mast cell activation markers, such as tryptase levels, have been associated with disease severity, exacerbations, and poorer outcomes. Monitoring mast cell activation markers assists in predicting disease exacerbations and guiding treatment strategies.^[31]^ In systemic mastocytosis, a disorder characterized by abnormal mast cell proliferation, specific mast cell markers like serum tryptase levels and other genetic mutations serve as prognostic indicators. Elevated serum tryptase levels correlate with disease burden and severity, aiding in assessing the prognosis and guiding therapeutic decisions.^[30]^ Mast cells’ involvement in neuroinflammatory conditions, such as multiple sclerosis and migraine headaches, suggests a potential prognostic role. Mast cell markers and their correlation with disease activity or severity may provide insights into the course of these conditions and guide treatment strategies.^[31]^ Monitoring mast cell activation and specific mast cell markers provides valuable insights into disease prognosis, aiding clinicians in assessing the severity of conditions, predicting outcomes, and optimizing patient management strategies. Continued research into mast cell markers and their prognostic implications holds promise for improving prognostic assessments and refining therapeutic interventions in diverse medical conditions.

## 6. Mast cell-mediated immune responses in HIV infection

Mast cells, while traditionally associated with allergic responses and host defense against pathogens, are increasingly recognized for their potential contributions to immune responses in HIV infection. Despite not being the primary target cells for HIV replication, mast cells reside in various tissues and mucosal surfaces, placing them in proximity to sites vulnerable to viral entry.^[34–37]^ Mast cells express various receptors, including CD4 and CCR5, which are involved in HIV entry. While mast cells are not productively infected by HIV, they might serve as a reservoir for the virus or facilitate viral uptake and transmission to other susceptible cells, including CD4 + T cells, through trans-infection mechanisms.^[38–40]^

Mast cells respond to HIV-related stimuli, such as viral proteins or immune mediators, by releasing an array of cytokines, chemokines, and inflammatory mediators. These released factors can modulate immune responses, influencing the activation state of neighboring immune cells and potentially contributing to the local immune microenvironment.^[41–45]^ Mast cell activation in response to HIV antigens or immune complexes can trigger the release of inflammatory mediators, contributing to local inflammation. Chronic activation and sustained release of these mediators might lead to immune dysregulation, potentially exacerbating HIV-associated inflammation and tissue damage.^[46–49]^ Mast cells can interact with other immune cells, such as dendritic cells, macrophages, and T cells, influencing their activation and function. These interactions might shape adaptive immune responses against HIV and impact the overall immune landscape during infection.^[50–52]^ Given their presence in mucosal tissues, mast cells might play a role in mucosal immunity against HIV. Their interactions with the mucosal immune system and potential involvement in modulating immune responses at these sites could impact HIV transmission and early viral dissemination.^[53–55]^ However, the precise contributions of mast cells to HIV pathogenesis and immunity are still being elucidated. Further research is warranted to clarify their specific roles, the consequences of their activation, and their potential as therapeutic targets in HIV infection. Understanding mast cell-mediated immune responses in HIV infection holds promise for uncovering novel insights into viral pathogenesis and immune modulation, potentially informing future strategies aimed at modulating immune responses and controlling HIV transmission or disease progression.

## 7. Clinical applications

The clinical applications of understanding mast cell biology and its relevance in various diseases, including HIV infection, hold significant promise for diagnostic, prognostic, and therapeutic advancements.^[56]^ Mast cell markers, such as tryptase levels in serum or tissue biopsies, can aid in diagnosing mast cell-related disorders like systemic mastocytosis. Monitoring mast cell activation markers may assist in diagnosing allergic diseases, assessing disease severity, and identifying triggers for allergic reactions.^[57]^ Mast cell markers have shown promise as prognostic indicators in different diseases. For instance, the density of mast cells within tumors has been associated with prognosis in certain cancers. Elevated mast cell activation markers in cardiovascular diseases or allergic disorders can predict disease severity and risk of adverse events.^[58]^ Assessing mast cell activation markers can help monitor responses to treatments in mast cell-related diseases. In allergic conditions, tracking changes in mast cell activation might guide treatment adjustments and evaluate the effectiveness of therapies.^[59]^ Understanding mast cell biology opens avenues for developing targeted therapies. For instance, in allergic diseases, drugs targeting mast cell mediators, such as histamine receptors or specific cytokines, can alleviate symptoms and improve patient outcomes. Modulating mast cell activation or function might offer therapeutic benefits in various diseases, including HIV infection. Research into targeting mast cells to influence immune responses may lead to novel immunomodulatory strategies in managing HIV/AIDS, potentially impacting disease progression or transmission.^[60]^ Mast cell markers might aid in personalized medicine approaches by providing insights into individual disease profiles and guiding tailored treatment strategies for patients with mast cell-related disorders or conditions influenced by mast cell activity.^[61]^ Continued research into mast cell biology, their involvement in various diseases, and their potential as therapeutic targets will likely expand the scope of clinical applications. Translating these insights into clinical practice could lead to more precise diagnostic methods, improved prognostic assessments, and innovative therapeutic interventions across a spectrum of diseases, including HIV/AIDS.

## 8. Future perspectives

The future perspectives regarding mast cells hold significant promise across several domains, encompassing research, diagnostics, therapeutics, and potential clinical applications. Further exploration of mast cell markers and the development of more sensitive and specific diagnostic tools could enhance the accuracy of diagnosing mast cell-related disorders. Advancements in imaging technologies might enable better visualization and quantification of mast cells in various tissues. Identification of novel mast cell-specific biomarkers and signatures associated with specific diseases or conditions could refine prognostic assessments and treatment monitoring. These biomarkers might offer insights into disease progression, therapeutic responses, and patient stratification.^[62]^ Advancements in understanding mast cell signaling pathways and their interactions with other immune cells may pave the way for targeted therapies. Precision therapies targeting specific mast cell mediators or receptors could lead to more effective and tailored treatments for mast cell-related diseases, including allergies and mastocytosis. Research into novel therapeutic agents, such as mast cell stabilizers or modulators, could lead to innovative treatments for mast cell-related disorders. Developing drugs targeting mast cell receptors or mediators might offer new avenues for disease management. Incorporating mast cell markers and insights into disease pathophysiology could advance personalized medicine approaches. Tailoring treatments based on individual mast cell profiles might improve therapeutic outcomes and minimize adverse effects in patients with mast cell-related conditions. Understanding mast cell interactions at mucosal sites and their role in mucosal immunity may contribute to the development of mucosal vaccines. Leveraging mast cells’ involvement in immune responses might inform novel vaccine strategies, including those aimed at preventing HIV transmission. Encouraging collaborations between immunologists, allergists, oncologists, infectious disease specialists, and researchers from various fields could facilitate a comprehensive understanding of mast cell biology. Interdisciplinary efforts might accelerate discoveries and translational applications in mast cell-related research. Continued research efforts, technological advancements, and collaborative endeavors in mast cell biology are pivotal for unlocking the full potential of mast cells in disease diagnosis, prognosis, and therapeutic interventions. Exploring these future perspectives holds immense promise for improving patient care and advancing the management of various diseases influenced by mast cell activity.

Table 1 shows mast cell markers and their diagnostic significance in HIV/AIDS, Table 2 shows prognostic significance of mast cell markers in HIV/AIDS, Table 3 shows diagnostic techniques for mast cell marker analysis in HIV/AIDS, Table 4 shows challenges in mast cell marker analysis in HIV/AIDS, Table 5 shows emerging mast cell markers in HIV/AIDS research and Table 6 shows future directions in mast cell marker research for HIV/AIDS (provided by the author).

**Table 1:** Mast cell markers and their diagnostic significance in HIV/AIDS patients.

Mast cell marker	Diagnostic significance
CD117 (c-kit)	Elevated levels correlate with increased mast cell proliferation and activation in HIV/AIDS patients.
Tryptase	Increased serum levels indicate mast cell activation and may reflect systemic inflammation and disease progression.
Chymase	Elevated levels are associated with tissue remodelling and fibrosis, contributing to HIV/AIDS-related complications.
CD203c	Increased expression on mast cells suggests their involvement in HIV/AIDS-related immune dysregulation.
CD68 (KP1)	Elevated expression in tissue macrophages may indicate mast cell recruitment and activation in HIV/AIDS pathogenesis

**Table 2:** Prognostic significance of mast cell markers in HIV/AIDS.

Mast cell marker	Prognostic significance
CD117 (c-kit)	High expression correlates with disease progression, increased viral load, and poor prognosis in HIV/AIDS.
Tryptase	Elevated levels are associated with HIV/AIDS-related complications, such as cardiovascular disease and neurocognitive impairment.
Chymase	Correlates with tissue fibrosis, which may contribute to organ damage and worsen prognosis in advanced HIV/AIDS.
CD203c	Altered expression is linked to immune dysfunction and may predict disease progression and poor outcomes.
CD68 (KP1)	Increased expression in tissue macrophages may indicate chronic inflammation and a poor prognosis in HIV/AIDS.

**Table 3:** Diagnostic techniques for mast cell marker analysis in HIV/AIDS.

Diagnostic technique	Description
Immunohistochemistry (IHC)	Detection of mast cell markers in tissue samples to assess their distribution and expression levels.
Flow cytometry	Quantification of mast cell marker expression on peripheral blood cells for diagnostic purposes.
ELISA	Measurement of soluble mast cell markers in serum or plasma samples to assess systemic levels.
PCR	Detection of mast cell marker gene expression in tissue or blood samples for diagnostic purposes.
Immunofluorescence	Visualization of mast cell markers in tissue sections to study their localization and interactions.

**Table 4:** Challenges in mast cell marker analysis in HIV/AIDS.

Challenge	Description
Heterogeneity of mast cell markers	Variability in expression levels and distribution of mast cell markers in different tissues and disease stages.
Sample availability and quality	Limited access to well-preserved tissue or blood samples, affecting the accuracy of mast cell marker analysis.
Interference from other cells	Mast cell marker expression may be influenced by interactions with other immune cells and cellular microenvironments.
Standardization of assays	Lack of standardized protocols and reference values for mast cell marker analysis complicates result interpretation.
Data interpretation	Complex interactions between mast cells and HIV/AIDS pathogenesis require careful interpretation of marker data.

**Table 5:** Emerging mast cell markers in HIV/AIDS research.

Emerging mast cell marker	Description
CD117 isoforms	Alternative splicing generates multiple isoforms of CD117, which may have distinct roles in HIV/AIDS pathogenesis.
Tryptase isoforms	Different isoforms of tryptase may exhibit varying enzymatic activities and functions in the context of HIV/AIDS.
Mast cell MicroRNAs	MicroRNAs regulate mast cell activation and function, and dysregulation may contribute to HIV/AIDS-related complications.
Mast cell metabolites	Metabolomic profiling of mast cell products may uncover novel biomarkers and therapeutic targets in HIV/AIDS research.

**Table 6:** Future directions in mast cell marker research for HIV/AIDS.

Research direction	Description
Targeted therapies	Development of mast cell-targeted therapies to modulate immune responses and mitigate HIV/AIDS-related complications.
Biomarker discovery	Identification of novel mast cell markers and biomarkers to improve diagnostic and prognostic assessment in HIV/AIDS.
Immunomodulatory effects	Investigation of mast cell-mediated immunomodulation and its implications for HIV/AIDS pathogenesis and treatment.
Precision medicine	Integration of mast cell marker analysis into precision medicine approaches for personalized HIV/AIDS management.
Therapeutic interventions	Exploration of mast cell-based interventions, including mast cell stabilizers and inhibitors, for HIV/AIDS treatment.

## 9. Conclusion

In conclusion, the evolving landscape of mast cell research presents a wealth of opportunities across diverse fields, spanning diagnostics, therapeutics, immunology, and personalized medicine. Mast cells, once primarily associated with allergic reactions, have emerged as pivotal contributors to various diseases, including cancers, allergic disorders, cardiovascular ailments, and potentially HIV infection. The multifaceted roles of mast cells, encompassing immune modulation, tissue repair, and interactions with other immune cells, underscore their significance in health and disease.

As mast cells continue to unveil their intricate roles in health and disease, harnessing their potential could pave the way for transformative advancements in medicine, offering tailored treatments, precision diagnostics, and innovative immunomodulatory strategies across a spectrum of conditions. The journey into the future of mast cell research holds immense promise, shaping the landscape of medical science and improving the lives of individuals affected by mast cell-related disorders.

## Author contributions

**Conceptualization:** Emmanuel Ifeanyi Obeagu.

**Methodology:** Emmanuel Ifeanyi Obeagu.

**Supervision:** Emmanuel Ifeanyi Obeagu.

**Visualization:** Emmanuel Ifeanyi Obeagu.

**Writing – original draft:** Emmanuel Ifeanyi Obeagu.

**Writing – review & editing:** Emmanuel Ifeanyi Obeagu.
